# Reverse innovation in global health systems: towards global innovation flow

**DOI:** 10.1186/1744-8603-9-36

**Published:** 2013-08-30

**Authors:** Shamsuzzoha B Syed, Viva Dadwal, Greg Martin

**Affiliations:** 1 Globalization and Health, c/o BioMed Central, 236 Gray’s Inn Road, 156 London WC1X 8HB, United Kingdom

## 

The global flow of knowledge, skills, and ideas has been a defining feature of human progress. Different regions and peoples have contributed to and, indeed, led innovation development at various times in human history. From Africa to Asia, Latin America, and the Middle East, the current body of knowledge on these diverse contributions to human science and medicine is expanding [1]. For example, written a thousand years ago in the Middle East, the Qanun fi-l-tibb (Canon of Medicine) of Ibn Sina is an immense encyclopaedia of medicine that served as the chief guide to medical science in Europe for over six centuries [2]. Prior to vaccination, eighteenth century Europeans were eager to learn about and adopt innovative ideas to combat smallpox, including through variolation, which was long practiced in Africa and Asia [3]. The current global use of artemisinin anti-malarials as a standard treatment saving millions of lives is based on knowledge harnessed from Chinese medicine [4]. Indeed, it is hard to imagine a world without such noteworthy contributions; the health systems of today represent the culmination of centuries of global innovation flow.

The development pdigm in recent history has chiefly focused on promulgating ideas and health systems solutions that have been developed in rich countries with the expectation that a grateful and deferential “South” blithely adopt them. In contrast, there has lately been a growing realization that the prodigal and often extravagant “North” has something to learn from innovations that emerge from resource-challenged settings. Out of necessity, poorer countries have had to rethink processes, interventions, and overall systems to ensure the best value for money is attained at every turn. In a time of global austerity there is a growing appreciation of the need for bidirectional exchange of ideas to be the new adage of global dialogue.

The view that businesses in “developed” countries could create opportunities from innovative products and services arising from emerging economies was highlighted by John Hagel III and John Seely Brown, through a term defined as “innovation blowback” [5]. Govindarajan and colleagues advanced this concept a few years later, coining the term “reverse innovation” to refer to any innovation “likely to be adopted first in the developing world before spreading to the developed world” [6]. Dominant business thinking posits a number of drivers behind the innovation imperative of resource-challenged developing countries, including affordability; use of ‘service ecosystems’; robust product development; creative application setting; and leapfrog technologies [7]. Since Hagel and Brown, a set of related terms, such as frugal and disruptive innovation, have accompanied the discourse on the subject. At the same time, the phenomenon of two-way flow of knowledge, ideas, and products has had significant bearing on the field of global health, a field that naturally interacts with both the medical and social sciences disciplines and has its fair share of interdisciplinary partnerships, networks, and collaborations. Only recently has attention turned to focusing on “developing” country collaborators and innovators as partners, rather than just potential consumers [8].

A growing group of leaders and practitioners see an emerging future in reverse innovation in global health systems, a broad trans-disciplinary movement which seeks to make use of low-income country health innovations within high-income country settings. In this regard, there is already some emerging evidence of key system-wide benefits that may be accrued by developed countries in partnering with developing countries. This emerging evidence spans all six WHO health system building blocks: health service delivery; health workforce; health information; products, vaccines, and technologies; financing and leadership & governance [9].

We initiated this special series in an attempt to harness the worldwide impetus on “developed-developing” country learning experiences and to start developing a robust knowledge base on the bi-directional flow of knowledge and innovations between low, middle, and high-income countries. We were delighted to discover that our initial call for papers generated immense and diverse levels of interest from all corners of the world. As a result, we decided to make this series an on-going collection. In this issue we embark on our journey by showcasing six prominent articles that offer a taste of this rapidly expanding area of inquiry.

Binagwaho et al. offer examples of numerous innovations from Rwanda to highlight the ways in which the global health community can leverage international partnerships for shared learning and improved health outcomes [10]. Jones et al. synthesize evidence on the impact of volunteering within health partnerships with low-income countries on health workforce development and service delivery in the United Kingdom [11]. Johnson et al. introduce readers to a pilot community health worker project in North Wales, which found inspiration from a Brazilian community health worker model— without doubt, a project to keep a close eye on [12]. Drawing on core concepts from business and innovation literature, De Passe and Lee propose a new model to help accelerate the flow of health solutions within the reverse innovation pipeline. Dandonoli explores the concept of “open innovation collaborations” through reflecting on work conducted in a maternal, newborn and child health initiative. Finally, Thunhurst examines the historical two-way innovation flow between “developed” and “developing” countries in public health systems and operational research.

The series on reverse innovation in global health systems is couched within, and intimately interconnected to, a broader global movement aimed at recognizing the real potential of low and middle-income countries in contributing to health system challenges globally. This movement traverses many borders and each of the above articles adds to the global pool of knowledge in various intersecting fields, including health, development, and social innovation. We are at a very early stage in our understanding of this phenomenon and a blueprint is required for us to achieve the ultimate aim of effective global innovation flow. The underlying concepts on the subject have started to be explored by advocates of developed-developing country partnerships through the promotion of bi-directional flow of knowledge, ideas, skills and innovation. Yet, fundamental obstacles to global innovation flow, too, remain strong. In this regard, three barriers stand out in particular: first, weak flow infrastructure; second, narrow-mindedness; and third, early failures.

Through this series, we aim to be a part of the response to these barriers. By systematically collecting evidence on the subject, we will build the leading repository of open access peer-reviewed publications on reverse innovation in global health systems—the “go to” place for those interested in this multidisciplinary field. We hope that global discussions that have been catalysed as a result of the series will also challenge and rethink traditional practice within global health systems, by not only highlighting the need for the open-mindedness in the “North” but also by encouraging the architects of new ideas in the “South” to confidently promote the adoption of their innovations abroad. These conversations will provide fuel to a global movement that has, for too long, gone unrecognized in dominant literature and public opinion. Finally and most importantly, our focus is to learn from, share, and critique successes and failures in order to build a truly robust evidence-base on the subject.

Reverse innovation in global health systems has the potential to contribute to the countless health challenges faced by populations across the world. Our ultimate destination – global innovation flow – may be decades away, but will certainly benefit from focused attention on how innovations in so-called “developing” countries inform health systems across the world.
